# Relationships Between Exposure to Different Gambling Advertising Types, Advertising Impact and Problem Gambling

**DOI:** 10.1007/s10899-021-10038-x

**Published:** 2021-05-31

**Authors:** André Syvertsen, Eilin K. Erevik, Daniel Hanss, Rune A. Mentzoni, Ståle Pallesen

**Affiliations:** 1 Department of Psychosocial Science, University of Bergen, P.O. box 7807, 5020 Bergen, Norway; 2 Norwegian Competence Center for Gambling and Gaming Research, University of Bergen, Bergen, Norway; 3 Department of Social Sciences, Darmstadt University of Applied Sciences, Darmstadt, Germany

**Keywords:** Gambling marketing, Gambling disorder, Advertising types, Advertising influence, Gambling promotion

## Abstract

People with gambling problems report more exposure and impact from gambling advertising, although less is known regarding the role of specific advertising types. Data on gamblers (*n* = 5830, 48.5% women, mean age = 44.27) was collected from a general population cross-sectional survey in Norway (32.7% response rate). We examined if problem gambling was associated with perceived advertising impact (on gambling involvement, awareness, and knowledge) or exposure (via internet, TV, retail outlet, newspaper, and direct advertising). We also investigated if advertising exposure was associated with advertising impact. ANOVAs revealed that problem gambling was associated with increased perceived advertising impact on gambling involvement (ω^2^ = 0.09, *p* < .001) and awareness of gambling (ω^2^ = 0.04, *p* < .001). Reported exposure to direct advertising increased linearly with problem gambling level (ω^2^ = 0.04, *p* < .001), whereas we found small/no differences in exposure to other types of advertising. Multiple regressions revealed that among advertising types, internet advertising was the strongest predictor of perceived advertising impact on gambling involvement (β = 0.1, *p* < .001). TV advertising was the strongest predictor of advertising impact on knowledge of gambling forms and operators (β = 0.28, *p* < .001) and awareness of gambling (β = .05, *p* < .05). Future studies should elucidate how different subtypes of internet advertising impact gambling involvement. Clinicians should assess clients’ experiences with direct advertising and devise interventions for coping. Researchers should be aware that internet and direct advertising allow for more tailored content compared to other advertising types.

## Introduction

Modern gambling advertising is becoming more tailored towards target groups in terms of content and types of communication (Binde, 2014; Newall et al., 2019). TV advertising may for example present high risk types of gambling such as casino games and contain content designed for men or women, specifically (Håkansson & Widinghoff, 2019). Gambling advertising is also prevalent in social media, allowing for tailored advertising such as posts containing hyperlinks to unique promotions and specific gambling objects, humorous content, and content difficult to identify as gambling advertising (Gainsbury et al., 2016; Thomas et al., 2015). Direct advertising such as emails, text messages or phone calls enable gambling operators to target individual gamblers and offer tailored forms of marketing (Russell et al., 2018; Syvertsen et al., 2020).

One group that seems to be more likely to be targeted by gambling advertising is people with gambling risk/problem gambling. Problem gambling involves, among other things, lack of control of gambling and harm caused by gambling, either to the individual or others (Ferris & Wynne, 2001). Gambling risk involves some gambling problems and harm, but to a lesser degree than problem gambling. Several studies have found that people with risk/problem gambling report increased exposure to gambling advertising (Clemens et al., 2017; Gavriel-Fried et al., 2010; Hanss et al., 2015). One reason of this is that those with gambling risk/problem gambling may be more attentive towards gambling advertisement (Gainsbury et al., 2016). Secondly, those with gambling risk/problem gambling may be exposed to more gambling advertisement due to the places they frequent (e.g., gambling sites) and gambling companies targeting advertisement directly towards them (Gainsbury et al., 2016).

Gambling advertising appears to impact all types of gamblers. Gambling advertising is associated with increased gambling engagement (Binde, 2014; Newall et al., 2019). Studies have found positive associations between gambling advertising exposure and increased gambling intention, which may be mediated by changes in attitudes towards gambling (Felsher et al., 2004; Hing et al., 2013; Lee et al., 2008). The impact of gambling advertising is further supported by findings suggesting that exposure to gambling advertising is positively associated with gambling frequency and gambling risk/problem gambling (Clemens et al., 2017; Gavriel-Fried et al., 2010; Hanss et al., 2015). People with problem gambling appear further to be more vulnerable to gambling advertising, as they have been found to be more likely to report that advertising triggers thoughts about gambling, increased intention to gamble, actual gambling, and increased risk-taking during gambling compared to those with non-problem gambling (Binde & Romild, 2019; Gainsbury et al., 2016; Hanss et al., 2015; Hing et al., 2018; Salonen et al., 2018). People with problem gambling may be more vulnerable to gambling advertising because they are more exposed to it and/or because they are particularly susceptible for the messages in gambling advertisement.

Specific types of advertising seem to be associated with specific types of impact. Receiving advertising emails is associated with increased betting intention and receiving text messages is associated with increased betting likelihood and size of bets (Russell et al., 2018). Content analysis of direct advertising shows that it commonly features promotional elements, such as bonus offers, boosted odds, and fixed percentages of losses back (Rawat et al., 2019). Promotional advertising, direct or indirect, may be especially impactful as it facilitates gambling more directly compared to brand-awareness advertising (Hing et al., 2018, 2019).

Recent developments in gambling advertising emphasize the need for research to account for different types of gambling advertising and their specific impacts. Identifying advertising-type-specific associations with gambling could inform priorities in terms of preventive measures, policy decisions, and special considerations in clinical work with people with problem gambling (Binde, 2009, 2014; Hing et al., 2014). In the present study, we examine the relationship between exposure to different advertising types and impact from gambling advertising, broken down on gambling risk/problem gambling. Advertising impact is defined as perceived changes in gambling involvement, awareness towards gambling, or knowledge about gambling forms and operators because of gambling advertisement (Hanss et al., 2015). The present study uses data from a recent general population survey conducted in Norway in 2019 (Pallesen et al., 2020). We posit the following hypotheses:

1. Gambling risk/problem gambling is positively associated with increased exposure to advertising.
  - The association between gambling risk/problem gambling and advertising exposure is stronger for internet and direct advertising than for TV, retail outlet, and newspaper advertising.
2. Gambling risk/problem gambling is positively associated with perceived advertising impact (involvement, awareness, and knowledge).
3. Advertising exposure is positively associated with perceived advertising impact.

Hypothesis 1 is based on findings that those with gambling risk/problem gambling report more exposure to gambling advertising (Clemens et al., 2017; Gavriel-Fried et al., 2010; Hanss et al., 2015). Hypothesis 1a is a secondary exploratory hypothesis, derived from the assumption that internet and direct advertising are more dependent upon an individual’s engagement with the advertising type or gambling overall compared to TV, newspaper, and retail advertising. That is, in the case of internet advertising, people with problem gambling may be more likely to follow gambling-related social media accounts and thus receive more gambling-related pop-ups and internet advertisements while browsing the internet. Likewise, direct advertising exposure could also depend on the individual’s level of engagement with gambling companies (Syvertsen et al., 2020). TV, newspaper, and retail advertising may offer less opportunity for such engagement. Hypothesis 2 is based on quantitative studies finding positive associations between gambling risk/problem gambling and advertising impact, as well as qualitative studies showing that people with problem gambling report that gambling advertising causes them to gamble more (Binde, 2009; Binde & Romild, 2019; Gainsbury et al., 2016; Hanss et al., 2015; Hing et al., 2014, 2018; Salonen et al., 2018; Syvertsen et al., 2020). Hypothesis 3 is based on previous studies finding positive associations between advertising exposure and gambling behavior or advertising impact (Clemens et al., 2017; Felsher et al., 2004; Hanss et al., 2015; Hing et al., 2013; Lee et al., 2008).

## Methods

### Participants and Procedure

We conducted a general population survey in fall 2019. In total, 30,000 individuals between ages 16 and 74 were randomly selected from the Norwegian Population Registry. The selected individuals first received an invitation by postal mail with information about the study and a web adress for completing the survey online. Those who did not answer online, received a reminder by mail combined with a paper based version of the survey. The original invitation was sent in August 2019, a first reminder in September 2019 and a final reminder in October 2019. The data collection ended on December 31st 2019. Participants were entered into a raffle of 200 gift cards, each valued at 500 NOK (approximately 49 €) and two Iphone X 256 GB smartphones.

Responses were deemed valid, if participants reported whether or not they had engaged in gambling during the last 12 months. This led to 9248 valid responses which translated to a response rate of 32.7%, after excluding 1676 invitations that had been returned due to invalid adresses and another 22 responses due to death, staying abroad, language difficulties or illness. The sample in the present study only included the participants who reported that they had engaged in gambling (*n* = 5830).

## Measures

### Demographic Information

Participants’ age and biological sex were collected from the population registry. The responses were weighted to adjust for discrepancies between the sample and the population in terms of age, biological sex and county. Age ranged from 16 to 74 years, with a mean age of 44.27 (*SD* = 15.89). There were 48.5% women (*n* = 2854). Place of birth was assessed with the question “Where were you born?” and included the following response alternatives: Norway (*n* = 5183), nordic country outside Norway (*n* = 152), Europe outside nordic countries (*n* = 259), Africa (*n* = 30), Asia (*n* = 136), North America (*n* = 25), South- or Central America (*n* = 30), and Oceania (*n* = 1).

### Canadian Problem Gambling Index (CPGI)

The CPGI is a validated measure of problem gambling (Ferris & Wynne, 2001). It consists of nine items, assessing problematic gambling behavior (four items) and negative consequences from gambling (five items) on a 4-point scale ranging from 0 (“never”) to 3 (“always”). Cronbach’s alpha for the nine items was 0.91 (*n* = 5805). Responses were grouped into non-problem (composite sum score of 0), low risk (1–2), moderate risk (3–7), and problem gambling (8–27), resulting in 4624 cases with non-problem (78.7%), 814 with low risk (13.9%), 286 with moderate risk (4.9%), and 126 with problem gambling (2.1%).

### Gambling Advertising Exposure Measures

Gambling advertising exposure was measured by five items. Each item asked participants about exposure during the previous 12 months to one of five different advertising types, including: TV, internet, newspapers, retail outlets, and direct advertising (e.g. text, email, phone calls). The five items were rated on a 5-point scale ranging from (1) “never”, (2) “less than one day each month”, (3) “approximately monthly”, (4) “approximately weekly”, and (5) “approximately daily”.

### The Effects of Gambling Advertising Questionnaire (EGAQ)

The original EGAQ contains four subscales assessing impact of gambling advertising (Derevensky et al., 2007, 2010). The current study used one original subscale containing five items along with four additional items that were formulated to capture additional impacts of gambling advertising. Exploratory and confirmatory factor analyses of the revised scale has revealed three factors: “involvement” (5 items), “awareness” (2 items), and “knowledge” (2 items) (Hanss et al., 2015). Example items are “I am more likely to gamble after seeing a gambling advertisement” (involvement), “I don’t pay attention to gambling advertisement” (awareness), and “gambling advertisement has increased my knowledge of gambling options” (knowledge). Items are rated on a 4-point scale ranging from (1) “strongly disagree”, (2) “somewhat disagree”, (3) “somewhat agree” and 4 (“strongly agree”).

We conducted a confirmatory factor analysis with IBM AMOS 25 on the three-factor structure that was found in Hanss et al. (2015). Maximum likelihood estimation using the unweighted sample of gamblers (*n* = 5830) was used. Measurement fit (χ^2^ = 548.07, *df* = 24, χ^2^/*df* = 22,84, p < 0.001, CFI = 0.96, RMSEA = 0.06, *CI* [0.057 to 0.066]) was acceptable. Correlations between latent factors were 0.65 for involvement and awareness, 0.35 for involvement and knowledge, and 0.19 for knowledge and awareness. Standardized regression weights of the latent factors on the observed variables ranged from 0.52 to 0.92. The results overall supported the factor structure reported by Hanss et al. (2015). Index variables were computed for each factor. A composite sum score was calculated for each factor so that higher scores indicate more influence from gambling advertising. Cronbach’s alpha value was 0.85 for involvement (*n* = 5890). Spearman–Brown values were 0.84 for knowledge (*n* = 5805) and 0.52 for awareness (*n* = 5825).

## Statistical Analysis

We examined Hypotheses 1 (association between risk and exposure), 1a (stronger association between risk and internet/direct advertising exposure, compared to other types of advertising), and 2 (association between risk and advertising impact) by conducting one-way ANOVAs on mean differences in gambling advertising impact and gambling advertising exposure among different risk categories of gamblers. Hochberg’s GT2 was used as post-hoc test due to unequal size of gambling risk categories. We examined Hypothesis 3 (association between advertising exposure and advertising impact) by conducting multiple regressions with advertising exposure for the different types as predictor variables and the advertising impact indices as dependent variables. Each multipe regression contained the same predictor variables (the five types of advertising exposure, age, biological sex and a dichtomous gambling problem variable). Inclusion of gambling problem allowed us to examine the impact of this while controlling for other key variables, which provided additional information for support or rejection of hypothesis 2 (risk and advertising impact). Gambling problem was dichotomized into non-problem/low risk (0) versus moderate risk/problem gambling (1). The analyses were performed with advertising impact on involvement, awareness and knowledge as dependent variables. Statistical assumptions for performing multiple linear regression were checked. We found no indication of multicollinearity, heteroscedasticity, or univariate outliers. Residuals were not perfectly normally distributed, however this “does not invalidate an analysis so much as weaken it” (Tabachnick & Fidell, 2014, p. 163). Multivariate outliers were examined by Mahalanobis distance scores against critical value adjusted for degrees of freedom (8 *df* = 26.13) which revealed 43 multivariate outliers. Ultimately, we decided to keep the cases as they constituted only a small percentage of the sample (0.7%) (Cohen et al., 2003).

Cases were excluded pairwise for multiple regressions and ANOVAs. Effect sizes were calculated in terms of omega squared (ω^2^) for one-way ANOVAs and adjusted *R*^2^ and β for multiple regressions (Fritz et al., 2012). One intepretation of ω^2^ and adjusted *R*^2^ effect sizes suggests that 0.04 constitutes a practically significant effect, 0.25 a moderate effect, and 0.64 a strong effect (Ferguson, 2009). For β, 0.2 constitutes a practically significant effect, 0.5 a moderate effect, and 0.8 a strong effect.

## Ethics

We conducted the study in accordance with the Helsinki Declaration and all participants provided informed consent. The study was approved by the Norwegian Centre for Research Data, project number 528056.

## Results

The most common type of gambling advertisement exposure was TV (*M* = 3.5, *SD* = 1.25), followed by retail outlets (*M* = 3.17, *SD* = 1.28), internet (*M* = 3.15, *SD* = 1.32), newspapers (*M* = 2.04, *SD* = 1.1), and direct advertising (*M* = 1.98, *SD* = 1.16). Differences in advertising type of exposure were broken down on gambling risk/problem gambling (see Table 1 with associated test statistics). We found a statistically significant effect for differences between groups for total advertising exposure, riskier gambling was associated with higher advertising exposure than less risky gambling, although the effect size was below practical significance. Likewise, we found non-significant and/or negligible effect sizes for the differences between groups for TV, internet, newspaper, and retail outlet advertising. This suggests that gamblers within different risk categories report practically similar amounts of gambling advertising exposure from TV, internet, newspapers, and retail outlets. These results counter Hypothesis 1 (i.e., those with risk/problem gambling will report more advertising exposure). However, exposure to direct advertising showed a statistically significant effect that was also practically significant, indicating that those with riskier gambling reported receiving more direct marketing. This provides some support for Hypothesis 1a (i.e., those with risk/problem gambling will report more exposure to direct and internet advertising).

**Table 1:** Mean differences in advertising exposure

Gambling risk/problem gambling														
Advertising exposure	Nonproblem			Low risk			Moderate risk			Problem			Welch *F*	ω^2^
	M	SD	SE	M	SD	SE	M	SD	SE	M	SD	SE		
Total advertising	2.72^a^	0.84	0.12	2.87^b^	0.82	0.03	3.06^c^	0.87	0.05	3.26^c^	0.84	0.08	(3, 398.40) = 32.8**	0.02
TV advertising	3.48	1.24	0.18	3.58	1.26	0.05	3.61	1.31	0.08	3.61	1.41	0.13	(3, 403.94) = 2.4	0.00
Internet advertising	3.05^a^	1.32	0.02	3.43^b^	1.25	0.04	3.68^c^	1.23	0.07	3,75^b^	1.31	0.12	(3, 404.74) = 47.4**	0.02
Newspaper advertising	2.05^a^	1.10	0.02	1.91^b^	1.07	0.04	2.06	1.19	0.07	2.35^c^	1.23	0.11	(3, 397.56) = 6.3**	0.00
Retail outlet advertising	3.15	1.27	0.02	3.22	1.30	0.05	3.33	1.35	0.08	3.43	1.28	0.12	(3, 397.45) = 3.6*	0.00
Direct advertising	1.87^a^	1.09	0.02	2.22^b^	1.26	0.05	2.61^c^	1.34	0.08	3.08^d^	1.29	0.12	(3, 391.31) = 72.7**	0.04

One-way ANOVAs conducted on mean differences in advertising exposure. Welch *F* statistics are reported because of unequal variances in gambling categories. Hochberg's GT2 used as post-hoc test due to unequal size of gambling risk categories. Means displayed in different superscript letters are significantly different at *p* < .05. For Welch *F*: *p < . 05, **p < .001

Within the entire sample, gambling advertising was reported to increase knowledge of gambling forms and operators (*M* = 2.95, *SD* = 0.88), but to a lesser degree to increase awareness towards gambling (*M* = 1.87, *SD* = 0.82) and gambling involvement (*M* = 1.71, *SD* = 0.69). Differences in self-reported impact were broken down on gambling risk/problem gambling (see Table 2 with associated test statistics). Statistically significant mean differences between groups were found for all three types of impact and exceeded practical significance for awareness and involvement impacts. The results support Hypothesis 2 (i.e., gambling risk/problem gambling is positively associated with perceived advertising impact). The largest effect was found for involvement. Here, reported impact increased with gambling risk/problem gambling, indicating that those with the riskiest gambling also reported the strongest impact from gambling advertising. A similar but weaker trend was found for awareness. Less variation between groups was found for impact on knowledge, although those with non-problem gambling reported statistically significant lower impact when compared to all other groups.

**Table 2:** Mean differences in advertising impact

Gambling risk/problem gambling																		
Advertising impact	Nonproblem				Low risk				Moderate risk				Problem				Welch *F*	ω^2^
	*M*	*SD*	*SE*		*M*	*SD*	*SE*		*M*	*SD*	*SE*		*M*	*SD*	*SE*			
Involvement	1.60^a^	0.62	0.01		1.96^b^	0.72	0.03		2.29^c^	0.77	0.05		2.71^d^	0.77	0.07		(3, 408.30) = 198.7*	0.09
Awareness	1.80^a^	0.81	0.01		2.02^b^	0.81	0.03		2.22^c^	0.81	0.05		2.65^d^	0.75	0.07		(3, 424.28) = 82.1*	0.04
Knowledge	2.90^a^	0.89	0.01		3.12^b^	0.80	0.03		3.14^b^	0.83	0.05		3.17^b^	0.92	0.08		(3, 424.74) = 23.2*	0.01

One-way ANOVAs conducted on mean differences in advertising impact. Welch *F* statistics are reported because of unequal variances in gambling categories. Hochberg's GT2 used as post-hoc test due to unequal size of gambling risk categories. Means displayed in different superscript letters are significantly different at *p* < .05. For Welch F: * *p* < .001

Multiple regression analyses were conducted to examine the relative contribution of different types of gambling advertising exposure and gambling problems on specific types of advertising impact. We also controlled for age and biological sex. The multiple regressions were statistically significant (see Table 3). Examination of the adjusted *R*^2^ values revealed practically significant effect sizes for knowledge and involvement impacts. The results provide partial support for Hypothesis 3 (i.e., advertising exposure is positively associated with perceived advertising impact) as some associations between advertising exposure types and impacts reached statistical significance.

**Table 3:** Multiple linear regressions of advertising impacts

Dependent variables: advertising impact factors														
Independent variables	Involvement					Awareness					Knowledge			
	*B*	*SE*	β	*t*		*B*	*SE*	β	*t*		*B*	*SE*	β	*t*
Constant	1.63	0.05		34.78**		1.65	0.06		28.11**		2.39	0.06		42.38**
Biological sex^b^	0.01	0.02	0.01	0.65		− 0.05	0.02	− 0.03	− 2.18*		0.04	0.02	0.02	1.79
Age	− 0.01	0.00	− 0.11	− 8.05**		0.01	0.00	0.01	0.82		− 0.01	0.01	− 0.23	− 17.35**
Gambling problems^a^	0.67	0.03	0.25	19.48**		0.51	0.04	0.16	11.75**		0.04	0.04	0.01	0.98
TV advertising	− 0.02	0.01	− 0.04	− 2.54*		0.03	0.01	0.05	3.27*		0.2	0.01	0.28	20.54**
Internet advertising	0.05	0.01	0.10	5.97**		0.02	0.01	0.03	1.47		0.09	0.01	0.13	8.35**
Newspaper advertising	0.01	0.01	0.02	1.18		0.02	0.01	0.03	1.94		-0.01	0.01	− 0.01	− 0.82
Retail outlet advertising	0.02	0.01	0.04	2.63*		0.01	0.01	0.01	0.47		0.05	0.01	0.07	5.03**
Direct advertising	0.02	0.01	0.04	3.00*		0.01	0.01	0.01	0.64		− 0.02	0.01	− 0.02	− 1.79
	adj. *R*^2^ = 0,117; *F*(8, 5681) = 94,848**					adj. R^2^ = 0,032; F(8, 5697) = 24,713**					adj. R^2^ = 0,220; F(8, 5677) = 201,653**			

^a^0 = nonproblem or low risk, 1 = moderate risk or problem gambling. ^b^women = 1, men = 2. *p < .05. ** *p* < .001

Examination of standardized βs in the model for involvement revealed that gambling problems had the strongest association to impact, compared to age, biological sex, and advertising exposure types variables. This indicates that those with gambling problems report higher gambling involvement due to gambling advertisement. Age was negatively associated with involvement, implying that younger people reported being more involved in gambling due to advertising. For advertising types, statistically significant effects were found for TV, internet, retail outlets, and direct advertising. The effects were small overall, with the strongest effect being found for internet advertising, followed by equal strengths of associations for TV, retail outlets, and direct advertising. The results indicate that exposure to these advertising types is associated with increased involvement in gambling, supporting Hypothesis 3. Surprisingly, a negative effect was found for TV advertising which indicates that increased exposure to TV gambling advertising is associated with less involvement in gambling, which runs counter to Hypothesis 3. However, examination of bivariate correlations for the variables included in the multiple regressions revealed a modest positive correlation (see Table 4), suggesting that the negative association in the multiple regression may be due to common variance with other predictors.

**Table 4:** Zero-order pearson correlation matrix for variables in multiple linear regressions of advertising impact

Correlations											
	Involvement	Awareness	Knowledge	Biological sex^b^	Age	Gambling problems^a^	TV advertising	Internet advertising	Newspaper advertising	Retail outlet advertising	Direct advertising
Biological sex^b^	0.06**	0.01	0.09**								
Age	−0.17**	−0.01	−0.28**	−0.04**							
Gambling problems^a^	0.29**	0.16**	0.06**	0.11**	−0.13**						
TV advertising	0.05**	0.08**	0.36**	0.10**	−0.02	0.02					
Internet advertising	0.18**	0.07**	0.35**	0.19**	−0.31**	0.11	0.45**				
Newspaper advertising	0.06**	0.06**	0.12**	0.08**	0.14**	0.03*	0.31**	0.39**			
Retail outlet advertising	0.09**	0.05**	0.20**	0.09**	0.02	0.04**	0.35**	0.37**	0.42**		
Direct advertising	0.13**	0.07**	0.12**	0.08**	−0.04**	0.18**	0.24**	0.36**	0.27**	0.25**	

^**^Correlation is statistically significant at *p* < .01. *Correlation is statistically significant at *p* < .05. ^a^0 = nonproblem or low risk, 1 = moderate risk or problem gambling. ^b^women = 1, men = 2

Examination of standardized βs in the model for knowledge revealed that TV advertising and age showed the strongest association with increased knowledge of gambling forms and operators. Among remaining advertising types, a similar albeit weaker association was observed for internet and retail outlet advertising. Age was inversely associated with knowledge impact, so that younger gamblers report being more knowledgeable due to advertising compared to older gamblers.

Examination of standardized βs in the model for awareness revealed statistically significant effects for biological sex, gambling problems, and TV advertising. There was a small negative effect for biological sex, suggesting that women experience higher awareness of gambling forms and operators due to advertising. Having gambling problems was associated with higher awareness impact. Likewise, increased exposure to TV advertising was associated with higher awareness impact.

## Discussion

In this study, we aimed to uncover differences in gambling advertising type-specific exposure among different groups of gamblers, including those with gambling risk and problem gambling. Differences in perceived impact from gambling advertising was then investigated. In addition, we investigated if specific types of advertising exposure were associated with specific types of perceived impact of gambling advertising.

The results provided marginal support for Hypothesis 1 (i.e., gambling risk/problem gambling is positively associated with increased exposure to advertising): ANOVAs revealed non-significant or significant but small differences between groups for total advertising as well as for TV, retail, internet, and newspaper advertising exposure. However, direct advertising exposure increased linearly with gambling risk severity, and this effect was practically significant. The results supported Hypothesis 2 (i.e., gambling risk/problem gambling is positively associated with perceived advertising impact): ANOVAs revealed statistically significant differences between groups on all types of impact, although the effect size for knowledge impact was below practical significance. Multiple regressions revealed statistically significant associations between gambling problems and involvement and awareness, but not knowledge. Hypothesis 3 (i.e., advertising exposure is positively associated with perceived advertising impact) is partially supported by the results: Exposure to TV advertising predicted higher awareness and knowledge impacts, but less involvement impact in the multiple regression (and opposite to the bivariate correlation). Internet advertising exposure predicted higher involvement and knowledge impact. Retail outlet advertising predicted higher involvement and knowledge impact. Direct advertising predicted higher involvement impact. No effects were found for newspaper advertising.

Overall, exposure to TV, internet and retail outlet advertising was high in the present sample. This is in line with previous general population studies in Norway, indicating that these types of advertising are ubiquitous and, in the case of TV advertising, have increased during recent years (Pallesen et al., 2016). Differences in mean internet advertising exposure were only statistically significant between those of no vs. any gambling risk. The upper bound of the confidence interval approached the maximum value for people with problem gambling which could indicate a ceiling effect. It is possible that clearer differences in advertising exposure between risk categories would emerge if additional response categories were added (e.g., “multiple times per day” in addition to “approximately daily”). Direct advertising was relatively infrequent among gamblers of no risk but became more frequent with increasing gambling risk/problem gambling. In terms of response categories, those with non-problem reported approximately monthly exposure while those with problem gambling reported approximately weekly exposure to direct advertising. These results are in line with qualitative studies revealing that people with problem gambling report extensive experiences with direct advertising (Hing et al., 2014; Syvertsen et al., 2020). These findings carry implications for clinical work (see below).

Gambling risk/problem gambling was associated with higher impact from gambling advertising. This is in line with previous research and can be interpreted as further support for the notion that people with problem gambling are more vulnerable to gambling advertising (Binde & Romild, 2019; Gainsbury et al., 2016; Hanss et al., 2015; Hing et al., 2018; Salonen et al., 2018). The largest effect size was found for involvement impact in the analysis of impact by risk category. Relatedly, gambling problems emerged as the strongest predictor of involvement impact in the multiple regression. This was also found in a previous study using the same measure (Hanss et al., 2015). The findings are notable as the involvement impact relates to gambling behavior, with advertising being perceived to increase interest and gambling intention, actual gambling, and risk taken during gambling. Influence on gambling behavior likely carries more risk of developing problem gambling compared to influence on awareness towards gambling or knowledge about gambling forms and operators. Those with gambling risk/problems may report more involvement impact due to being exposed to different and more effective types of marketing, such as promotional advertising including free credits, boosted odds, bonuses, and loss returns (Hing et al., 2018, 2019).

Different types of gambling advertising were associated with different types of advertising impact. TV gambling advertising was the most frequently experienced type of advertising, which could explain why exposure to this type had the strongest positive association with increased knowledge of gambling forms and operators due to advertising. TV advertising was also associated with increased awareness of gambling. TV advertising may contain more brand-awareness content, compared to other types of advertising which may explain why it was associated with increased knowledge and awareness impact.

Internet advertising exposure showed the strongest association with increased gambling involvement compared to the other advertising types. Internet advertising differs from other types of advertising by being more interactive; gamblers may click on website ads and social media posts to directly access both promotional offers and gambling opportunities (Gainsbury, 2012; Gainsbury et al., 2016; Thomas et al., 2015). The interactive nature of internet advertising may explain why this type of advertising were more strongly related to involvement impact. In addition, internet advertising was associated with knowledge impact.

Retail outlet advertising was associated with increased gambling involvement and knowledge. Point-of-sale advertising draws recipients’ attention toward gambling while simultaneously providing availability of gambling opportunity, possibly facilitating more impulsive gambling. Recall of point-of-sale lottery advertising has been positively associated with intention to gamble on lotteries (Felsher et al., 2004; Monaghan et al., 2008). No effect was found for newspaper advertising in the present study. In Norway, only licensed gambling operators are allowed to market gambling products (Rossow & Hansen, 2016). This restriction is more successfully enforced for retail outlet and newspaper advertising, while restrictions on TV, internet, and direct advertising are circumvented by unregulated gambling operators by broadcasting from abroad. Norwegian licensed gambling advertising is also regulated in terms of content, and any effects/lack of effects regarding retail outlet and newspaper advertising could be influenced by this.

Direct advertising was also associated with gambling involvement impact. Direct advertising frequently includes promotional advertising which, as noted above, may be more effective than advertising focusing on brand awareness or knowledge (Rawat et al., 2019; Russell et al., 2018). Of all advertising types examined in the present study, direct advertising exposure varied most between risk categories of gamblers. The association between direct advertising and involvement impact is worrisome when viewed in light of findings that those with riskier gambling receive considerably more direct advertising and experience more advertising impact overall. Despite internet advertising showing the strongest association with involvement impact in general, direct advertising might pose a greater challenge for those with gambling risk/problems when viewing the above together.

Younger gamblers reported higher gambling involvement due to gambling advertising, which is in line with previous studies (Clemens et al., 2017; Derevensky et al., 2010; Hanss et al., 2015). We also found that young age was associated with higher knowledge of gambling forms and operators due to advertising. Online popular culture is embedded with gambling content, and young people have a larger online presence (King et al., 2010; Thomas et al., 2012). Young men have also reported that sports betting advertising target their lifestyle and identity, with a messaging that normalizes gambling involvement (Deans et al., 2017). In the current study, biological sex differences were, however, observed for awareness impact only: Women reported more awareness of gambling due to advertising compared to men. These findings differ from a previous study in which men reported more involvement and knowledge impact compared to women, whereas no biological sex difference was observed for awareness impact (Hanss et al., 2015).

### Strengths and Limitations

A major strength of the present study is that it comprises a large and nationally representative sample. While there are previous gambling advertising studies with nationally representative samples studying gambling advertising (e.g. Binde & Romild, 2019; Hanss et al., 2015; Salonen et al., 2018), the present study provides more detail to the impacts of specific advertising types.

A limitation to the study is the relatively low response rate of 32.7%. Unfortunately, low response rates are not uncommon for these types of studies, and a general trend of falling response rates in epidemiological surveys has been observed (Galea & Tracy, 2007). A comparable Norwegian general population survey achieved 43.6% in 2013 and a Finnish general population survey achieved the more similar 36% in 2016 (Hanss et al., 2015; Salonen et al., 2018). We used several strategies to increase response rate, including having online as well as mail response modes, offering reminders with paper versions of survey and using raffles for compensation (Edwards et al., 2009; Stedman et al., 2019).

Advertising types may be differentiated to a larger degree than what was done in the present study. Gambling advertising occurs in a myriad of settings. Aspects of advertising not considered in the current study include advertisement during sporting events, via celebrity endorsements and on promotional products (Lopez-Gonzalez & Griffiths, 2018; Monaghan et al., 2008). Greater nuance could also be achieved within internet advertising by separating social media and internet website advertising (Thomas et al., 2015).

It should also be noted that the current study was cross-sectional and relied upon self-report measures. The cross-sectional nature of the present study prevents us from concluding regarding directionality and causality. However, the study was strengthened by having participants report on the perceived causal effect of advertisement on their gambling involvement, awareness, and knowledge. It is a strength that the current study uses an alternative measure of impact compared to previous studies that examined associations between advertising exposure and gambling behavior measures (Binde, 2014). Still, self-report measures are limited by recall bias in general, and in the case of advertising research it can be argued that advertising is likely to subtly influence gambling behavior and attitudes over time, and individuals may not be fully aware of how they have been impacted, and from which type of advertising, thereby limiting the accuracy of information (Heath et al., 2006). It should also be noted that people assume other people are more susceptible to gambling advertising compared to themselves—the third-person effect (Youn et al., 2000). This further supports that self-report measures may underestimate the true impact of gambling advertising.

### Study Implications and Conclusion

In this study, we found that, among advertising types, internet advertising was the strongest predictor of increased gambling involvement due to advertising. We also found that direct advertising exposure was more prevalent among those with riskier gambling. Both findings should be expanded upon in future research. We examined degree of exposure rather than content. This can be further studied by content analysis of gambling advertisements typically featuring in each advertising type. For example, previous content analysis of TV advertising in Sweden suggests that casino gambling advertising is frequent and content analysis of direct advertising suggests that it contains more promotional types of advertising than other types of gambling advertisement (Håkansson & Widinghoff, 2019; Rawat et al., 2019). We suggest that researchers who conduct content analysis bear in mind the potential for gambling operators to personalize both internet advertisements (through “cookies”) and direct advertisements. Advertising recipients’ level of engagement with the gambling operator and/or their level of risk should be taken into the account. An interview study found that those with problem gambling received promotional offers and special gifts through direct advertising that were highly tailored, suggesting that it is likely that content differs among gamblers according to engagement (Syvertsen et al., 2020). By researching such forms of advertisement through signing up to gambling sites and analyzing routine emails one may fail to capture the types of advertisement that are experienced by more engaged gamblers.

Direct advertising may be relatively common for those with problem gambling. Clinicians should include questions during assessment covering experiences with amount, type, and impact of direct advertising. Clients with problem gambling may find this type of advertising hard to resist, as it targets them directly and may contain tailored offers. Treatment plans may thus include strategies to cope with gambling urges elicited by direct advertising.

Increased regulation of gambling advertising has been associated with lower problem gambling rates (Planzer et al., 2014). Still, the fact that advertising types have different and unique characteristics makes it difficult to apply successful universal regulations. For instance, the personal nature of direct advertising makes this form of advertising especially hard to monitor. Likewise, internet advertising may also be difficult to monitor and regulate as it can take covert forms, e.g., social media personalities promoting gambling through streaming platforms and social media posts without adequate labeling. Gambling researchers should consequently widen their view of what constitutes gambling advertising in the future, if we are to better capture the ways people are influenced by gambling advertisements.

## Data Availability

Study data is available upon request.

## Code Availability

SPSS syntax code for analyses is available upon request.

## Declarations

### Other

A report has previously been published in Norwegian based on the survey which includes some analyses of gambling advertising data (Pallesen et al., 2020). The current manuscript differs substantially from the report, here we provide new group-based analyses, multivariate analyses, and a conceptualization of the advertising impact measure.
